# Risk Factors for Fatal Pulmonary Hemorrhage following Concurrent Chemoradiotherapy in Stage 3B/C Squamous-Cell Lung Carcinoma Patients

**DOI:** 10.1155/2018/4518935

**Published:** 2018-11-01

**Authors:** Erkan Topkan, Ugur Selek, Yurday Ozdemir, Ali A. Besen, Ozan C. Guler, Berna A. Yildirim, Huseyin Mertsoylu, Alper Findikcioglu, Ozgur Ozyilkan, Berrin Pehlivan

**Affiliations:** ^1^Baskent University Medical Faculty, Department of Radiation Oncology, Adana, Turkey; ^2^Koc University, School of Medicine, Department of Radiation Oncology, Istanbul, Turkey; ^3^The University of Texas, MD Anderson Cancer Center, Department of Radiation Oncology, Houston, TX, USA; ^4^Baskent University Medical Faculty, Department of Medical Oncology, Adana, Turkey; ^5^Baskent University Medical Faculty, Department of Thoracic Surgery, Adana, Turkey; ^6^Bahcesehir University Medical Faculty, Department of Radiation Oncology, Istanbul, Turkey

## Abstract

We aimed to identify the fatal pulmonary hemorrhage- (FPH-) related risk factors in stage 3B/C squamous-cell lung carcinoma (SqCLC) patients treated with definitive concurrent chemoradiotherapy (C-CRT). Medical records of 505 stage 3B/C SqCLC patients who underwent 66 Gy radiotherapy plus 1-3 cycles of concurrent chemotherapy with available pretreatment thoracic computerized tomography scans were retrospectively analyzed. Primary end-point was the identification of FPH-related risk factors. Examined factors included the basal patient and tumor characteristics with specific emphasis on the tumor cavitation (TC) status, tumor size (TS) and cavitation size (CS), tumor volume and cavitation volume (TV and CV), relative cavitation size (RCS = CS/TS), and relative cavitation volume (RCV=CV/TV). FPH emerged in 13 (2.6%) patients, with 12 (92.3%) of them being diagnosed ≤12 months of C-CRT. All FPHs were diagnosed in patients with TC (N=60): group-specific FPH incidence: 21.6%. TC (P<0.001) was the unique independent factor associated with higher FPH risk in multivariate analysis. Further analysis limited to TC patients exhibited the RCV>0.14 (37.5% versus 11.1% for RCV≤0.14; P<0.001), major RCS group [31.0% versus 19.0% for minor versus 0% for minimum RCS; P=0.008), and baseline hemoptysis (26.3% versus 13.6% for no hemoptysis; P=0.009) as the independent risk factors for higher FPH incidence. FPH was an infrequent (2.6%) complication of C-CRT in stage 3B/C SqCLC patients, but its incidence increased to 37.5% in patients presenting with TC and RCV>0.14. Diagnosis of >90% FPHs ≤12 months of C-CRT stresses the importance of close and careful follow-up of high-risk patients after C-CRT for multidisciplinary discussion of possible invasive preventive measures.

## 1. Introduction

Survival advantage exhibited by phase III randomized controlled trials set the concurrent chemoradiotherapy (C-CRT) as the standard treatment decision for inoperable stage III non-small-cell lung cancer (NSCLC) patients [1, 2]. However, the established superiority of C-CRT over RT alone and sequential- or split-course CRT modalities undoubtedly came at the cost of notably increased normal tissue complication rates, particularly the RT-induced esophagitis and pneumonitis [3, 4].

Another serious but underestimated complication of RT or C-CRT in NSCLC is fatal pulmonary hemorrhage (FPH) with an incidence rate of 1.5-3.5% for all patients [5, 6]. However, this rate may increase up to 36% in cavitating squamous-cell lung cancers (SqCLC) [5, 6]. FPH has likewise been documented in NSCLCs undergoing endobronchial brachytherapy [7, 8], stereotactic body RT [9, 10], and antiangiogenic therapies [11, 12]. Interestingly, despite its life-threatening nature, to date, FPH-related risk factors following CRT have been studied in only a single study of 583 stage 2-3 NSCLC patients by Ito et al. [6]. The overall FPH incidence was 2.1% in this study, with SqCLC histology and tumor cavitation (TC) size being the significant associates of FPH. However, rendering thorough interpretation of the outcomes difficult, the authors provided no information about the total and per fraction doses of RT, exact type of CRT, and chemotherapy details, either of which may significantly alter the FPH rates. Moreover, decreasing the statistical power, the SqCLC cohort that has the highest risk for TC and FPH incidences constituted only 34.7% of the entire study population.

With reference to the absence of large exclusive SqCLC series, present retrospective study was conducted to further investigate the FPH-related risk factors following definitive C-CRT in a large cohort of consecutively treated stage 3B/C SqCLC patients.

## 2. Patients and Methods

### 2.1. Study Population

An institutional database search was performed to identify all patients who underwent C-CRT between January 2007 and December 2014 for stages 3B/C SqCLC according to American Joint Committee on Cancer (8th ed.) and met the following criteria: age of 18-80 years; available diagnostic chest computerized tomography (CT); ^18^F-fluorodeoxyglucose positron emission CT (PET-CT); Eastern Cooperative Oncology Group (ECOG) performance of 0-1; available pre-C-CRT brain magnetic resonance images, treatment charts, and hospital computerized datasets of RT and chemotherapy; at least 1 concurrent chemotherapy cycle administered; and no prior RT/chemotherapy histories. Patients presented with malignant pleural/pericardial effusion, inadequate pulmonary, cardiac, renal, or hepatic functions, and blood count/chemistry and those who received antiangiogenic therapies for relapses after C-CRT were excluded. The study was approved by the institutional review board before collection of any patient information.

### 2.2. Concurrent Chemoradiotherapy

All patients were treated with 3-dimensional conformal (3D-CRT) or intensity-modulated RT (IMRT). Target volume definition and treatment technique for RT and organ at risk dose restrictions utilized here were as previously described by Topkan et al. elsewhere [13]. Briefly, all patients received a total dose of 66 Gy RT in 33 fractions and 1 to 3 cycles of Cisplatin plus one of Vinorelbine, Docetaxel/Paclitaxel (Taxanes), or Etoposide. Standard supportive and symptomatic care measures were administered as indicated.

### 2.3. Fatal Pulmonary Hemorrhage Definition

FPH was defined as pulmonary hemorrhage that leads to inevitable fatality within 24 hours of its onset despite any type of intervention, excluding the causes related with proved tumor progression, infection, or trauma.

### 2.4. Assessment of Tumor Cavitation

TC was defined as presence of an air-containing cavity within the primary tumor mass on the pretreatment chest CT scans, which has been proposed to be a significant risk factor for FPH after C-CRT [6]. Therefore, the baseline chest CT scans of all patients were assessed by an experienced radiation oncologist on cavitating lung tumors (E.T) in order to determine the presence/absence of TC and delineate its borders on coregistered diagnostic and planning CT scans. If TC was multiloculated in the TV, all separate TC volumes were combined and considered as one CV. The TC presence status, largest sizes and volumes of the primary tumor and TC, and their relative ratios were evaluated as potentially relevant baseline tumor characteristics which may interact with FPH diagnosis.

### 2.5. Statistics

We primarily aimed to identify the incidence rate of FPH and related risk factors after definitive C-CRT in stage 3B/C SqCLC patients. Evaluable risk factors included the age, gender, ECOG performance, smoking history and duration, tumor stage and location, baseline hemoptysis, >5% weight loss status at past 6 months, baseline anemia and TC status, largest sizes and volumes of the primary tumor and TC and their relative ratios, RT technique, and concurrent chemotherapy protocol and cycles.

As the largest diameters of the TC and primary tumor may not always reflect the respective true volumes because of significant variations in their shapes and remaining two dimensions, we created a novel volumetric methodology to classify TCs, whereas Ito's classification was utilized only for comparative analyses [6]. Accordingly, we delineated and measured the tumor (TV) and TC (TCV) volumes separately for each patient by utilizing the diagnostic chest CT scans and defined the relative cavitation volume (RCV) as the TCV/TV ratio for further analyses. Likewise, the largest primary tumor (TS) and TC (TCS) sizes were measured and the relative cavitation size (RCS) was calculated for each patient as the TCS/TS ratio for Ito's TC classification: minimum: RCS<0.25, minor: RCS≥0.25 but <0.50, and major: RCS≥0.50.

Mean, median, and ranges were used for the quantitative variables, whereas frequency distributions were used to describe the categorical variables. Frequency distributions were compared by using Chi-square test, Student's t-test, Pearson's exact test, or Spearman's correlations. Receiver operating characteristic (ROC) curve analysis was utilized to identify potential cut-off values for continuous variables, such as the RCS and RCV, which may stratify patients into distinctive FPH risks groups. Fisher's exact test and logistic regression test were used for univariate and multivariate analyses, respectively. Multivariate analyses included only the variables exhibiting significance in univariate analyses. A two-sided P-value <0.05 was considered significant.

## 3. Results

Present database search identified a total of 505 stage IIIB/C SqCLC patients who met the protocol inclusion criteria. Baseline demographics and treatment characteristics were as depicted in Table 1. Of all patients, 16.2% were geriatric (>70 years). Smoking history was present in 93.9% of patients with a median smoking duration of 28 pack/years (range: 0-132). The Cisplatin-Vinorelbine doublet was the most commonly utilized chemotherapy (49.8%), and 75.1% of patients were able to receive all 3 prescribed chemotherapy courses during the RT. Baseline hemoptysis, WL>5% at past 6 months, and anemia were evident in 29.9%, 21.8%, and 35.6% of cases, respectively. TC was diagnosed in 60 (11.8%) patients.

At a median follow-up time of 28.3 months (range: 24.1- 32.5), FPH was diagnosed in 17 (3.4%) patients. However, 13 (2.6%) of them were judged to be true FPHs, as progressive primary tumors invading the major vessels were demonstrable in remaining 4 cases. All FPHs were diagnosed in 60 individuals with TC, accounting for an overall FPH incidence rate of 21.6% in this group. Median time from C-CRT to FPH diagnosis was 4.4 months (range: 2.3-13.1), with 8 (61.5%), 4 (30.8%), and 1 (7.7%) cases being diagnosed within 6, 6 to 12, and >12 months of C-CRT, respectively.

Results of univariate analyses discovered the central tumor location (r^2^=0.23; P=0.026), basal hemoptysis (r^2^=0.38; P=0.032), and TC (r^2^=0.91; P<0.001) as the significant correlates of higher FPH risk in the entire study population (Table 2). Outcomes of multivariate analyses limited to these covariates discovered the TC (P<0.001) as the unique factor to retain its independent significant association with FPH risk (Table 2).

**Figure 1:**
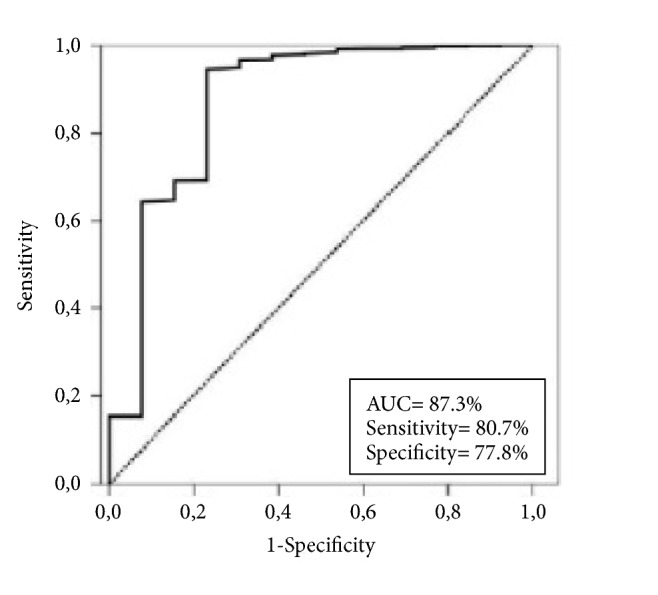
Outcomes of receiver operating characteristic curve analysis for the association between the relative cavitation volume and fatal pulmonary hemorrhage incidence risk.

In order to identify specific group(s) with the highest FPH risk, we restricted our further analyses to patients presenting with TC (N=60), as all FPHs were diagnosed in this group (Table 3). Before univariate analysis, we first searched for the availability of significant cut-off(s) for TS and TCS, TV, TCV, RCS, and RCV in TC patients which may stratify them into distinctive FPH risk groups. For this purpose, we performed ROC curve analysis, results of which revealed only a significant cut-off value for RCV at 0.14 point (area under the curve=87.3%; sensitivity: 80.7%; specificity: 77.8%) with no further identifiable cut-offs for other variables (Figure 1). Therefore, the median values were utilized for these variables in further analyses. Then, RCSs were classified as minimum (N=10; 16.7%), minor (N=21; 35.0%), and major (N=29; 48.3%) according to Ito's methodology [6]. The major RCS group appeared to have the highest FPH risk (31.0%) followed by the minor (19%) and minimum (0%) RCS groups (P= 0.008). Lastly, we dichotomized the TC patients into two groups according to their RCVs: Group 1 (N=36): RCV≤0.14 and Group 2 (N=24): RCV>0.14. Comparative analysis demonstrated that the risk of FPH was significantly higher in Group 2 compared to Group 1 (37.5% versus 11.1%; P<0.001). Among other factors, only the presentation with baseline hemoptysis appeared to be significantly associated with higher FPH risk (26.3% versus 13.6%; P=0.007) (Table 3). Results of multivariate analysis limited to Ito's groups, RCV groups, and hemoptysis status revealed that each factor retained its independent significant association with FPH risk, with RCV grouping being the strongest stratification factor according to the related multivariate P-values (Table 3).

## 4. Discussion

Results of our present large retrospective study in 505 stage 3B/C SqCLC demonstrated that the overall FPH risk after definitive C-CRT was 2.6%. TC was found to be the unique independent factor to predict FPH risk in the whole study population as all FPHs (N=13) were diagnosed in TC patients (N=60) with an increased FPH risk of 21.6% in this cohort. Resultant limitation of the analyses to TC cohort additionally discovered the presenting hemoptysis, Ito's major TC class, and RCV>0.14 as the significant predictors of higher FPH risk in this particular group, with RCV>0.14 being the strongest factor among them.

The primary goal of our present research was to determine the key factors that may prove useful in identification of patients with the highest FPH risk after definitive C-CRT. Either of the rapid tumor regression, bronchial and/or vessel wall necrosis, or vascular endothelial abnormalities after thoracic RT/C-CRT may cause bronchovascular fistula. By extension, it is rational to postulate RT and to a larger extent C-CRT as potential causes of FPH with regard to the proven roles of RT-induced bronchovascular fistula and vascular abnormalities in the FPH pathogenesis [14–16]. However, excluding the single study by Ito et al. [6], all previous studies focused on the risk factors in NSCLC patients undergoing antiangiogenic therapies, such as bevacizumab, sunitinib, and sorafenib [17–19], and demonstrated a clear association between these agents and increased FPH risk mainly in non-SqCLCs. Despite the fact that FPH emerges in up to 50% NSCLC patients treated with endobronchial brachytherapy [7, 8, 16], investigations that concentrated on its incidence and related risk factors after RT/C-CRT are scarce, with a reported 1.5-3.5% incidence range [5–7, 20]. However, this rate may increase up to 36% in cavitating SqCLCs [5, 6]. Hence, the accessible basic and clinical evidence motivated us to conduct this present study to identify the candidates who might require treatment customization in follow-up by further addressing the incidence and risk factors of FPH particularly in exclusive SqCLC patients who underwent standard C-CRT for stage 3B/C disease with no antiangiogenic therapies at any phases of their treatment course.

The first important finding of our study was the exhibition of a very strong and independent association between the TC presence and FPH risk (N=13) after C-CRT, such that although overall incidence of FPH was 2.6% in the whole study population, this value increased to 21.6% in TC cohort with no FPH diagnosis (0%) in the non-TC cohort (P<0.001). The exact mechanism of increased FPH rates in TC cohort has not been clarified yet, but vascular invasion by the tumor cells and related intratumoral ischemia have previously been reported to be common features of cavitating SqCLCs [21], which cause FPH by disruption of the abnormal tumor vessels via secretion of several transcription factors and angiogenic cytokines, such as the hypoxia-inducible transcription factors and vascular endothelial growth factors [22]. Whatever the exact cause of FPH is, the 2.6% FPH incidence reported here accords well and confirms the respective 2.3% and 2.1% incidence rates previously reported in Phermanbucq's [20] and Ito's [6] CRT studies. Moreover, our present findings that suggest the TC as the unique factor to be associated with increased FPH risk do not only lend support for Ito's observation of all 3 FPHs in their SqCLC subgroup with TCs but also clearly set the SqCLC plus TC combination as the major risk factor for FPH emergence after definitive C-CRT as well.

Because all 13 FPHs were diagnosed in 60 patients with TC, our secondary analyses limited to TC population revealed 3 additional important findings in this particular group: (1) hemoptysis as a significant associate of FPH, (2) confirmation of the utility of Ito's 3 laddered RCS-based FPH risk stratification methodologies, and (3) demonstration of large RCV>0.14 as a novel independent volumetric predictor of higher FPH risk. To begin with, compared with the former study by Ito et al. [6] which could not reveal a notable interaction between the baseline hemoptysis and FPH risk, the significant association between the baseline hemoptysis and higher FPH risk demonstrated here is a new finding. Although it is difficult to assign this discrepancy to solid reasons, the differences between the two statistical methodologies may be explanatory. In Ito's study, the authors limited their analysis to the whole population which included different histologies and stages, with no respect to their TC status. In contrast, because the tumoral blood vessel erosion is a common cause for hemoptysis and TC particularly in the locally aggressive SqCLC [23], we further analyzed the interaction between the baseline hemoptysis and TC status in our exclusive stage 3B/C SqCLC patients presenting with TC. Although further validation is needed, the present strong association demonstrated in a statistically more powered large SqCLC cohort suggests that the authors may have probably missed the chance for reaching a statistically significant outcome on this particular issue.

Considering Ito's RCS and our novel RCV grouping systems, our results did not only lend support for Ito's system to some extent but additionally demonstrated that the grouping of TC patients according to RCV cut-off of 0.14 was the strongest predictor of higher FPH risk in this patients' group (37.5% for RCV>0.14 versus 11.1% for RCV≤0.14; P<0.001). Present methodology is notably different from the one utilized by Ito et al. [6]. Revealed from the manuscript, Ito's team used arbitrary cut-offs at 0.25 and 0.50 points rather than using those obtained from more objective analytic methods, such as the ROC curve analysis. In fact, because we could not define a significant cut-off for RCS in the ROC curve analysis, we believe that our confirmatory results for RCS should still be interpreted with caution and readdressed in further studies. Comparably, present 3-dimensional volumetric methodology and use of undoubtedly more objective ROC curve analysis results appear to be more confidential than Ito's arbitrarily stratified unidimensional metric methodology. Because both the tumors and the TCs are naturally 3-dimensional volumetric structures, utilization of unidimensional largest diameters as the end-point measures will usually carry the risks for over- or underestimation of the outcomes, as this methodology does not care for the other determinants of volumetric calculations, namely, the other two dimensions and the shape of the volumes in interest. In contrast, representing a replicable and more reliable methodology, the volumetric measurements utilized in this present study care for both of the shapes and all three dimensions of the individual TV and TCV in a simultaneous manner.

The last essential result of the current study was the diagnoses of the respective 8 (61.5%) and 12 (92.3%) of all 13 FPHs within 6 and 12 months of the C-CRT. Unfortunately, rendering the comparisons between the two studies impossible, Ito's group did not provide any information about the interval between the CRT and FPH diagnoses [6]. However, Phernambucq et al. reported 2 FPHs at 1 month of C-CRT [20]. Dingemans et al. in phase I study of individualized, isotoxic accelerated C-CRT reported 1 FPH at 4 months of C-CRT [24]. Likewise, Llandau et al. reported 3 FPHs at 1, 4.5, and 14 months of C-CRT in their phase 1/2 trial of isotoxic dose-escalated C-CRT [25]. Altogether, these results suggest the FPH as an early event after C-CRT and recommend the careful close follow-up of high-risk patients, at least for the first 12 months of C-CRT. Such an approach may prevent FPH-related deaths in appropriately selected high-risk patients by means of timely use of preventive measures, such as planned post-C-CRT surgery in resectable peripheral tumors regardless of their nodal status or percutaneous embolotherapy/bronchial artery embolization [26, 27].

The present study has drawbacks. First, common to any single-institutional retrospective analysis, some unpredictable biases may have affected our results. Second, despite the overall large cohort size, diagnosis of all FPHs solely in the TC group comprising only 60 patients limited our ability to stratify these patients into further risk groups by utilizing recursive partitioning analysis methodology according to the accompanying significant predictors of FPH: baseline hemoptysis and RCV status. However, despite these limitations, this is the first study to identify the factors associated with significantly increased FPH risk after a relatively homogenous C-CRT protocol in a large cohort of exclusive stage 3B/C SqCLC patients who never received antiangiogenic therapy during any phase of treatment.

## 5. Conclusion

Results of this single institutional large cohort study clearly demonstrated that the presence of pretreatment TC was the unique independent predictor of relatively infrequent but life-threatening FPH following definitive C-CRT in stage 3B/C SqCLC patients. Furthermore, the analysis restricted to TC cohort additionally exhibited that RCV>0.14, with a FPH incidence of 37.5%, was the most notable risk factor for FPH emergence during the post-C-CRT period. Finally, diagnosis of vast majority of all FPHs (92.3%) in the first 12 months of C-CRT is strongly suggestive of close and careful follow-up of such patients against this fatal complication for multidisciplinary discussion of possible invasive preventive measures such as planned post-C-CRT surgery or percutaneous embolotherapy/bronchial artery embolization.

## Figures and Tables

**Table 1:** Baseline demographics and treatment characteristics.

**Variable**	**All Patients** **(n=505)**
Median age, y (range)	65.1 (32-79)

Age group, n (%)	
≤70 years	423 (83.8)
>70 years	82 (16.2)

Gender, n (%)	
Male	352 (69.7)
Female	153 (30.3)

ECOG performance, n (%)	
0	205 (40.6)
1	300 (59.4)

Smoking history, n (%)	
Absent	31 (6.1)
Present	474 (93.9)

Median smoking duration, pack/y	28 (0-132)

Weight loss, n (%)	
≤5%	395 (78.2)
>5%	110 (21.8)

^a^Anemia, n (%)	
Absent	325 (64.4)
Present	180 (35.6)

Hemoptysis, n (%)	
Absent	354 (70.1)
Present	151(29.9)

Stage, n (%)	
3B	275 (54.5)
3C	230 (45.5)

Tumor location	
Central	224 (44.4)
Peripheral	281 (55.6)

Median largest tumor size, cm (range)	5.1 (2.4-9.8)

Median tumor volume, cm^3^ (range)	50.8 (19.3-149.4)

Chemotherapy, n (%)	
CV	251 (49.8)
CT	226 (44.7)
CE	28 (5.5)

Chemotherapy cycles, n (%)	
1	35 (6.9)
2	91 (18.0)
3	379 (75.1)

RT technique, n (%)	
3D-CRT	286 (56.7)
IMRT	219 (43.3)

Tumor cavitation, n (%)	
Absent	445 (88.1)
Present	60 (11.9)

^a^Hemoglobin: <130 g/dL for men and <120 g/dL for women.

ECOG: Eastern Cooperative Oncology Group; CV: Cisplatin + Vinorelbine; CT: Cisplatin + Taxane; CE: Cisplatin + Etoposide; 3D-CRT: 3-dimensional conformal radiotherapy; IMRT: intensity-modulated radiotherapy.

**Table 2:** Uni- and multivariate correlates of fatal pulmonary hemorrhage.

**Variable**	**Patients** **(N=505)**	**FPH** **(N=13)**	**Univariate P-value**	**Multivariate** **P-value**
Age, y (%)				
≤70	423 (83.8)	11 (2.6)	0.81	-
>70	82 (16.2)	2 (2.4)		

Gender, n (%)				
Male	352 (69.7)	9 (2.5)	0.78	-
Female	153 (30.3)	4 (2.6)		

ECOG performance, n (%)				
0	205 (40.6)	5 (2.4)	0.86	-
1	300 (59.4)	8 (2.7)		

Smoking history, n (%)				
Absent	31 (6.1)	1 (3.2)	0.78	-
Present	474 (93.9)	12 (2.5)		

Median smoking duration, (pack/y)				
<28	251 (49.7)	6 (2.4)	0.96	-
≥28	254 (50.3)	7 (2.8)		

Weight loss, n (%)				
≤5%	395 (78.2)	8 (2.0)	0.21	-
>5%	110 (21.8)	5 (4.5)		

^a^Anemia, n (%)				
Absent	325 (64.4)	8 (2.5)	0.85	-
Present	180 (35.6)	5 (2.8)		

Hemoptysis, n (%)				
Absent	354(70.1)	7 (2.0)	0.032	0.011
Present	151(29.9)	6 (4.0)		

Stage, n (%)				
3B	275 (54.5)	7 (2.5)	O.98	-
3C	230 (45.5)	6 (2.6)		

Tumor location, n (%)				
Central	224 (44.4)	9 (4.0)	0.026	0.14
Peripheral	281 (55.6)	4 (1.4)		

^b^Median largest tumor size, n (%)				
≤5.1 cm	215 (42.6)	4 (1.9)	0.25	-
>5.1 cm	290 (57.4)	9 (3.1)		

^b^Median tumor volume, n (%)				
≤50.8 cm^3^	248 (49.1)	5 (2.0)	0.48	-
>50.8 cm^3^	257 (50.9)	8 (3.1)		

Chemotherapy, n (%)				
CV	251 (49.8)	6 (2.4)	0.69	-
CT + CE	254 (50.2)	7 (2.8)		

Chemotherapy cycles, n (%)				
1	35 (6.9)	1 (2.9)	0.77	-
2-3	470 (18.0)	12 (2.6)		

RT technique, n (%)				
3D-CRT	286 (56.7)	8 (2.8)	0.80	-
IMRT	219 (43.3)	5 (2.3)		

Tumor cavitation, n (%)				
Absent	445 (88.1)	0 (0)	<0.001	<0.001
Present	60 (11.9)	13 (21.6)		

^a^Hemoglobin: <130 g/dL for men and <120 g/dL for women.

^b^Median values were used, as receiver operating characteristic analysis did not reveal significant cut-off.

FPH: fatal pulmonary hemorrhage; ECOG: Eastern Cooperative Oncology Group; CV: Cisplatin + Vinorelbine; CT: Cisplatin + Taxane; CE: Cisplatin + Etoposide; 3D-CRT: 3-dimensional conformal radiotherapy; IMRT: intensity-modulated radiotherapy.

**Table 3:** Uni- and multivariate correlates of fatal pulmonary hemorrhage in patients with tumor cavitation.

**Variable**	**Patients** **(N=60)**	**FPH** **(N=13)**	**Univariate P-value**	**Multivariate** **P-value**
^a^Median TS, n (%)				
<5.1 cm	27 (45.0)	6 (22.2)	0.68	-
≥5.1 cm	33 (54.0)	7 (21.2)		

^a^Median TV, n (%)				
≤58.9 cm^3^	26 (43.3)	5 (19.2)	0.57	-
>58.9 cm^3^	34 (56.7)	8 (23.4)		

^a^Median CS, n (%)				
< 2.6 cm	28 (46.7)	6 (21.4)	0.86	-
≥ 2.6 cm	32 (53.3)	7 (21.9)		

^a^Median CV, n (%)				
< 22.3 cm^3^	27 (45.0)	5 (18.5)	0.23	-
≥ 22.3 cm^3^	33 (55.0)	8 (242		

^a^Median RCS, n (%)				
< 0.46	31 (51.7)	5 (16.1)	0.09	-
≥ 0.46	29 (48.3)	8 (27.6)		

Ito's RCS group, n (%)				
Minimum (≤0.25)	10 (16.7)	0 (0)	0.006	0.008
Minor (≥0.25 but <0.50)	21 (35.0)	4 (19.0)		
Major (≥0.5)	29 (48.3)	9 (31.0)		

RCV group, n (%)				
≤0.14	36 (55.0)	4 (11.1)	<0.001	<0.001
>0.14	24 (45.0)	9 (37.5)		

Hemoptysis, n (%)				
Absent	22 (36.7)	3 (13.6)	0.007	0.009
Present	38 (63.3)	10 (26.3)		

^b^Median values were used, as receiver operating characteristic analysis did not reveal significant cut-off.

FPH: fatal pulmonary hemorrhage; TS: tumor size; TV: tumor volume; CS: cavitation size; CV: cavitation volume; RCS: relative cavitation size (largest tumor diameter/largest cavitation diameter); RCV: relative cavitation volume (cavitation volume/tumor volume).

## Data Availability

The data used to support the findings of this study are available from the corresponding author upon request.
